# Transcriptome and Anthocyanin Profile Analysis Reveals That Exogenous Ethylene Regulates Anthocyanin Biosynthesis in Grape Berries

**DOI:** 10.3390/foods14142551

**Published:** 2025-07-21

**Authors:** Min Liu, Boyuan Fan, Le Li, Jinmei Hao, Ruteng Wei, Hua Luo, Fei Shi, Zhiyuan Ren, Jun Wang

**Affiliations:** College of Food Science and Engineering, Shanxi Agricultural University, Taigu 030801, China

**Keywords:** ethylene, anthocyanin, grape, metabolome, transcriptome

## Abstract

Anthocyanins are important phenolic compounds in grape skins, affecting the color, oxidation resistance, and aging ability of red wine. In recent years, global warming has had a negative effect on anthocyanin biosynthesis in grape berries. Ethylene serves as a crucial phytohormone regulating the development and ripening processes of fruit; however, the specific molecular mechanism and the regulatory network between ethylene signaling and the anthocyanin biosynthesis pathway remain incompletely understood. In this study, 400 mg/L ethephon (ETH) solution was sprayed onto the surface of grape berries at the lag phase (EL-34), and the changes in anthocyanin-related genes and metabolites were explored through transcriptomic and metabolomic analysis. The results showed that ETH treatment increased Brix and pH in mature berries. In total, 35 individual anthocyanins were detected, in which 21 individual anthocyanins were enhanced by ETH treatment. However, the anthocyanin profile was not affected by exogenous ethylene. Transcriptomics analysis showed that there were a total of 825 and 1399 differentially expressed genes (DEGs) 12 h and 24 h after treatment. Moreover, key structural genes in the anthocyanin synthesis pathway were strongly induced, including *VvPAL*, *VvCHS*, *VvF3H*, *VvF3′5′H*, *VvDFR* and *VvUFGT*. At the maturity stage (EL-38), the expression levels of these genes were still higher in EHT-treated berries than in the control. ETH treatment also influenced the expression of genes related to hormone biosynthesis and signal transduction. The ethylene biosynthesis gene (*VvACO)*, ethylene receptor genes (*VvETR2*, *VvERS1* and *VvEIN4*), ABA biosynthesis gene (*VvNCED2*), and ABA receptor gene (*VvPYL4*) were up-regulated by ETH treatment, while the auxin biosynthesis gene (*VvTAA3*) and seven genes of the auxin-responsive protein were inhibited by exogenous ethylene. Meanwhile, ETH treatment promoted the expression of the sugar transporter gene (*VvEDL16*) and two sucrose synthase genes (*VvSUS2* and *VvSUS6*). In EHT-treated berries, 19 MYB and 23 ERF genes were expressed differently compared with the control (*p* < 0.05). This study provides the theoretical foundation and technical support for the regulation of anthocyanin synthesis in non-climacteric fruit.

## 1. Introduction

Anthocyanins in grape skins are water-soluble natural pigments, which form anthocyanosides with sugar moieties via glycosidic bonds [1]. The content and type of anthocyanins not only affect the color, oxidation resistance and aging ability of red wine, but also influence its flavor and mouthfeel by interacting with other phenolic compounds [2]. The major anthocyanins found in grapes can be classified into five principal categories: delphinidin, cyanidin, petunidin, peonidin, and malvidin [3]. The anthocyanin biosynthesis pathway involves two kinds of genes: structural genes and regulatory genes. Structural genes encode the enzymes responsible for anthocyanin synthesis, including phenylalanine ammonia lyase (PAL), chalcone synthase (CHS), chalcone isomerase (CHI), flavanone 3-hydroxylase (F3H), dihydroflavonol reductase (DFR), anthocyanidin synthase (ANS), and UDP-glucose: flavonoid 3-O-glucosyltransferase (UFGT). Regulatory genes modulate the spatiotemporal expression of these structural genes [4]. The MYB transcription factors are important regulators in anthocyanin biosynthesis. In red grape, *VvMybA1* and *VvMybA2* were active, but they were not active in white grape, thereby inhibiting *UFGT* transcription [5]. Wang et al. (2021) found that ethephon (ETH) treatment for 12 days significantly up-regulated anthocyanin structural genes (*VvPAL*, *Vv4CH*, *VvCHS*, *VvCHI*, *VvF3H*, *VvUFGT*) and regulatory genes (*VvMYBA1*, *VvMYBA2*, *VvMYBA3*) [6]. Qin et al. (2024) revealed that VvMYB3 competitively bound to VvMYC1, disrupting the formation of the VvMYBA1-VvMYC1-WD40 (MBW complex), and inhibiting anthocyanin synthesis [7].

Ethylene serves as a crucial phytohormone regulating the development and ripening processes of fruit. Grapes are typically classified as non-climacteric fruit because they lack the characteristic ethylene surge and respiratory peak compared with climacteric fruit [8]. Extensive research demonstrated that exogenous ethylene treatment could promote anthocyanin accumulation in grape berries. ETH treatment enhanced the expression of PAL, C4H, CHS, CHI, F3H, F3’5’H and UFGT, leading to the increase in anthocyanin content in grape berries [6,9]. Ethylene treatment of Sangiovese grapes enhanced pectin methyl esterase and β-glucosidase activity in berry skins, resulting in wine with elevated levels of flavonols, anthocyanins, flavan-3-ols and stilbenes [10]. ETH application accelerated anthocyanin accumulation in Cabernet Sauvignon, but did not affect the weight and diameter of the berries [11,12]. Furthermore, the ethylene treatment duration exhibited an inverse relationship with anthocyanin content. In Red Cesanese grapes, anthocyanin content increased with a 15 h ethylene treatment, but decreased with a 36 h treatment [13].

In recent years, global warming has introduced new challenges to existing viticultural regions. It was demonstrated that high temperature during grape ripening decreased anthocyanin content at maturity [14,15]. As a key hormone, ethylene has important effects on anthocyanin synthesis, but the specific molecular mechanism and the regulatory network between ethylene signaling and the anthocyanin biosynthesis pathway remain incompletely understood. In this study, through transcriptomic and metabolomic analysis, we explored the changes in anthocyanin-related genes and metabolites in grape berries after ethylene treatment. This study could help us better understand the role of ethylene in anthocyanin biosynthesis in non-climacteric fruit.

## 2. Materials and Methods

### 2.1. Plant Materials

The experiment was carried out in 2023 in the Grape Germplasm Repository of the Pomology Institute of Shanxi Agricultural University (Taigu, China, 112.5° E, 37.4° N). The self-rooted Cabernet Sauvignon (*Vitis vinifera* L.) vines colonized in 2012 were used and trained in a modified vertical shooting system. The spacing between grapevines was 0.8 m, and the row spacing was 2.5 m, with a north–south orientation.

### 2.2. Field Treatments and Sampling

The experimental design followed a completely randomized approach with triplicate replicates, each containing ten plants per treatment. The modified E-L system was employed for phenological stage documentation [16]. At EL-34 (30 July), 400 mg/L ETH solution (0.1% *v*/*v* Tween 80) was sprayed on the surface of the grape berries. The control group was sprayed with distilled water containing Tween 80.

Fifty berries for each replicate of the treatment group (T) and the control group (CK) were randomly collected on EL-35 (3 August), EL-36 (9 August), EL-37 (9 September), and EL-38 (3 October) for detection of the ripening parameters. After 12 h and 24 h of treatment, 100 berries for each of the T and CK groups were collected, frozen in liquid nitrogen, and labeled as T12, T24, CK12 and CK24, to be used for RNA-seq analysis. When the grapes were mature (EL-38), 100 berries for each group were collected, frozen and used for metabolomic analysis.

### 2.3. Detection of the Ripening Parameters

During the EL-35–EL-38 period, grape berries were collected and their diameter and hundred grain weight were measured. The grape juice produced by manual pressing was used to measure Brix, pH and total acid content according to the method of OIV [12].

### 2.4. Anthocyanin Profile Analysis

Freeze-dried samples were homogenized using a Retsch MM 400 mixer mill. Aliquots of 50 mg powdered material were subjected to sequential extraction with 0.5 mL of methanol/water/hydrochloric acid (799:200:1, *v*/*v*/*v*) solution. The extraction procedure involved vortex mixing (10 min), ultrasonic-assisted extraction (10 min), and centrifugation (12,000× *g*, 4 °C, 3 min). After two extraction cycles, we combined the supernatants and filtered them through 0.22 μm PTFE before LC-MS/MS.

UPLC separation was performed on an ACQUITY BEH C18 column (100 mm × 2.1 mm, 1.7 μm) (Waters, Milford, MA, USA) using an ExionLC™ AD system (SCIEX, Framingham, MA, USA). The UPLC analysis utilized a mobile phase of water and methanol (both modified with 0.1% formic acid) with the following gradient: 0 min/95% aqueous, 6 min/50%, 12 min/5%, 14 min/95% (2 min hold). The analysis was performed at 0.35 mL/min flow rate with 40°C column temperature and 2 μL injections.

Analyses were carried out using a QTRAP^®^ 6500 (SCIEX, Framingham, MA, USA) mass spectrometer with positive-ion ESI and Analyst^®^ 1.6.3 control software. ESI conditions were optimized to: 550 °C source temperature, 5.5 kV spray voltage, and 35 psi curtain gas [17]. The qualitative analysis of mass spectrometry data was performed by constructing NWDB (Metware Database) based on standard compounds (Table S1). Quantitative analysis was conducted using the Multiple Reaction Monitoring (MRM) mode on a triple quadrupole mass spectrometer. Total anthocyanin content was the cumulative amount of all individual anthocyanins.

### 2.5. RNA-Seq Analysis

Total RNA was purified from berry powder using RNAprep Pure Plant Plus Kit (Tiangen, Beijing, China), followed by Illumina NovaSeq paired-end sequencing (2 × 150 bp) at Novogene (Beijing, China). Poly-T magnetic bead-enriched mRNA was reverse-transcribed into cDNA using M−MuLV Reverse Transcriptase (Thermo Fisher, Waltham, MA, USA). Libraries were constructed with size selection (370–420 bp, AMPure XP, Beckman Coulter, Indianapolis, IN, USA), PCR amplification (Phusion High-Fidelity DNA Polymerase, Thermo Fisher, Waltham, MA, USA), and quality control (Bioanalyzer 2100, Agilent, Santa Clara, CA, USA).

Quality-filtered reads were mapped to the *Vitis vinifera* reference genome (URGI) using HISAT2 (v2.0.5) with default parameters. Transcript abundance was normalized as FPKM value. Analysis of differentially expressed genes (DEGs) was performed with DESeq2 (v1.20.0, *p* < 0.05), followed by functional enrichment analysis of GO terms and KEGG pathways [18].

### 2.6. Quantitative Real-Time PCR Analysis

Total RNA was isolated from grape skins using Plant Total RNA Extraction Kit (BioTeke, Beijing, China). Then 500 ng of total RNA was reverse transcribed into cDNA using Vazyme HiScript II Q RT SuperMix Kit. Quantitative real-time PCR (qRT-PCR) was performed as previously described [18]. The reference gene *VvActin* was used for normalization. All reactions were performed in technical triplicates. Gene-specific primer sequences were provided in Table S2.

### 2.7. Data Statistics and Analysis

Independent samples t-test was performed using SPSS version 26.0 to evaluate the significance of differences between the samples of the T and CK groups (*p* < 0.05). Partial least square discriminant analysis (PLS-DA) were performed by Metabo-Analyst (http://www.metaboanalyst.ca/MetaboAnalyst/faces/home.xhtml, accessed on 1 April 2025). All figures were generated using GraphPad Prism 10.

## 3. Results

### 3.1. The Effect of ETH Treatment on the Ripening Parameters of Grapes

**Figure 1:**
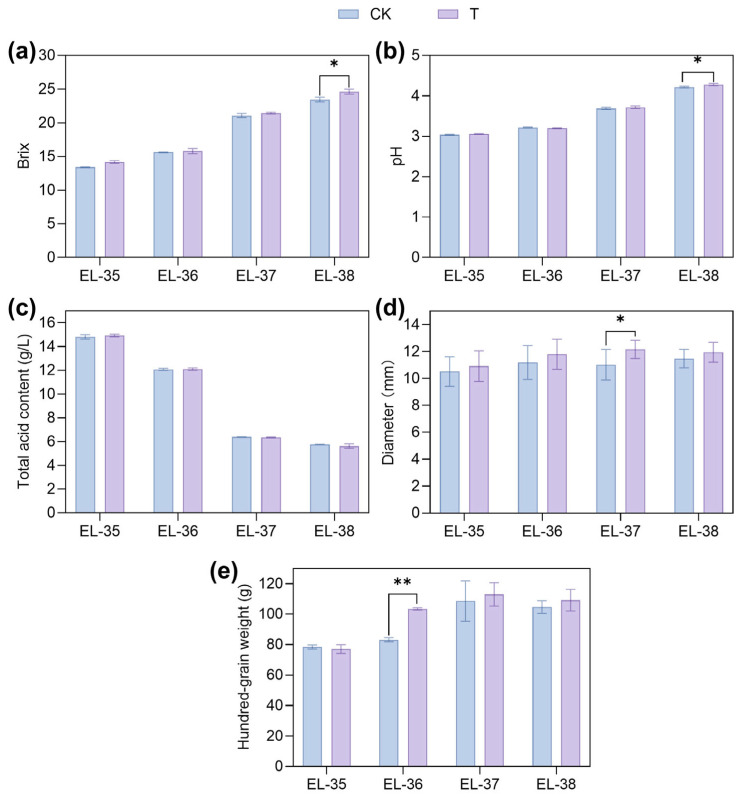
The effect of ETH treatment on (**a**) Brix, (**b**) pH, (**c**) total acid content, (**d**) diameter and (**e**) hundred-grain weight of grape. CK indicated the control group, and T indicated the ETH treatment group. One asterisk (*) and two asterisks (**) indicate significant differences at the levels of *p* < 0.05 and *p* < 0.01, respectively.

As the grapes matured, the diameter, hundred-grain weight, Brix and pH of the berries gradually increased (Figure 1a,b), while the total acid content showed a downward trend (Figure 1c). The diameters and hundred-grain weights in the T group were higher than those in CK group, but they were not significantly different at EL-38 (Figure 1d,e). In ETH-treated berries, Brix and pH were higher compared with the control at EL-38 (Figure 1a,b). However, the total acid content in the T and CK groups did not show a significant difference (Figure 1c).

### 3.2. Quantification of Individual Anthocyanins in Grape

**Figure 2:**
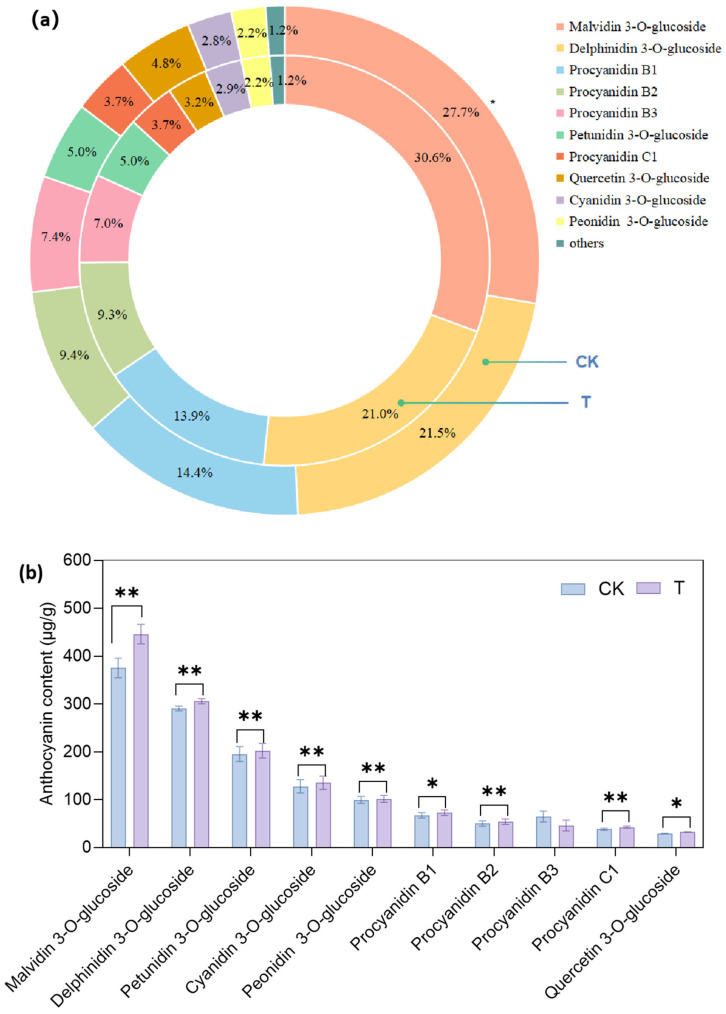
The proportions (**a**) and contents (**b**) of 10 dominant individual anthocyanins in grape berries. CK indicates the control group, and T indicates the ETH treatment group. One asterisk (*) and two asterisks (**) indicate significant differences at the levels of *p* < 0.05 and *p* < 0.01, respectively.

In the T and CK groups, 35 identical anthocyanins were detected, including 6 cyanidins, 5 delphinidins, 5 malvidins, 2 pelargonidins, 6 peonidins, 5 petunidins and 4 procyanidins (Table S3, Figure S1), indicating that exogenous ethylene had no effect on the anthocyanin profile in grape skins. The ETH treatment significantly increased the total anthocyanin content and the levels of 21 individual anthocyanins in grape skins (Table S3). As shown in Figure 2a, 10 dominant individual anthocyanins accounted for 98.8% of the total anthocyanin content. Among them, malvidin 3-O-glucoside was the most abundant, and its proportion in the T group (30.6%) was significantly higher than that in the CK group (27.7%) (Figure 2a). The proportions of the other nine individual anthocyanins in the treated berries was not different compared with the control (Figure 2a). From Figure 2b, we found that the contents of nine individual anthocyanins (malvidin 3-O-glucoside, delphinidin 3-O-glucoside, procyanidin B1, procyanidin B2, procyanidin B3, petunidin 3-O-glucoside, procyanidin C1, cyanidin 3-O-glucoside, peonidin 3-O-glucoside) were increased significantly in the ETH-treated berries. In a word, ETH treatment enhanced the anthocyanin content in grape skins, but did not influence its profile.

### 3.3. Transcriptomic Analysis of Grapes After ETH Treatment

**Figure 3:**
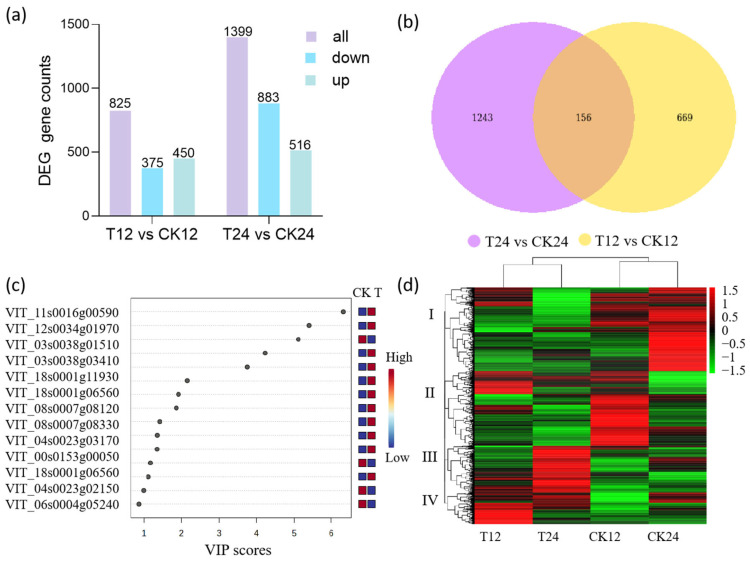
Transcriptome analysis of grape berries. (**a**) The counts of differentially expressed genes (DEGs). (**b**) Venn diagram of DEGs. (**c**) PLS-DA analysis of the transcriptomes. T indicates grape samples after ETH treatment, and CK indicates control samples without ETH treatment. (**d**) Heatmap visualization of genes. T12 and T24 indicate grape samples collected at 12 h and 24 h after ETH treatment, while CK12 and CK24 indicate control samples collected at the corresponding time points without ETH treatment.

To gain insights into the molecular mechanism underlying anthocyanin biosynthesis effected by ethylene, a transcriptomics analysis was conducted. In this study, a total of 31,330 genes were found in the grapes. In the T12 group, there were a total of 825 DEGs compared with CK12, including 375 down-regulated and 450 up-regulated genes (Figure 3a). In the T24 group, 1399 genes were expressed differently compared with CK24, in which 883 genes were down-regulated, and 516 genes up-regulated (Figure 3a). In the groups of T12 and T24, there were 156 co-expressed genes, which might play key roles in response to ethylene (Figure 3b, Table S4).

To further explore the effect of ETH on the expression of these co-expressed genes, a partial least squares discriminant analysis (PLS-DA) was conducted. Using the variable importance in the projection (VIP), we selected the important genes that contributed to separating the ETH treatment and the control (Figure 3c). In our study, genes with a VIP value above three included VIT_11s0016g00590 (Pectinesterase inhibitor 3), VIT_12s0034g01970 (Cupin), VIT_03s0038g01510, VIT_03s0038g03410 (NAC domain-containing protein 6), VIT_18s0001g11930 (pathogenesis-related thaumatin-like protein 3.5). Except for VIT_03s0038g01510, the other four genes were strongly induced by ETH treatment (Figure 3c, Table S4).

In this experiment, the hierarchical clustering method was employed to a cluster of 2068 DEGs based on their FPKM values after Z-score normalization, and the results are shown in the heatmap (Figure 3d). The color scale represents the normalized expression levels, with red indicating higher expression and green indicating lower expression. The dendrogram on the left represents the clustering of genes, and four groups of genes (I, II, III, IV) are clearly distinguishable. Meanwhile, the dendrogram at the top represents the clustering of samples. T12 and T24 were classified into the same cluster, and CK12 and CK24 were classified into another distinct cluster, suggesting that the ETH treatment had a significant impact on the gene expression pattern in grape berries.

**Figure 4:**
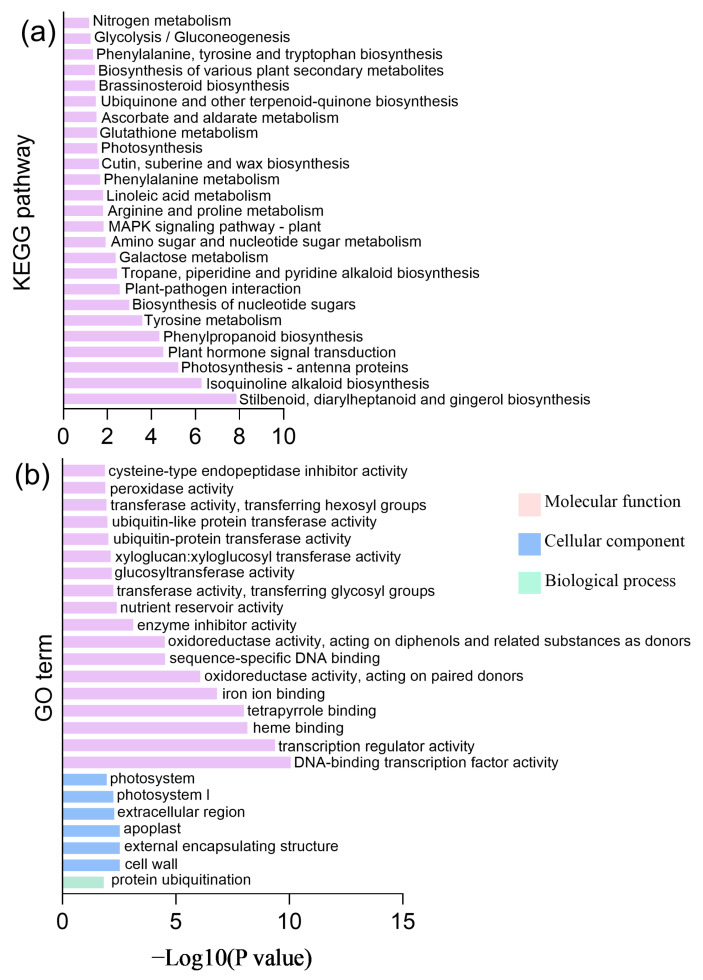
KEGG (**a**) and GO (**b**) enrichment analysis of DEGs.

To explore the biological functions of DEGs, GO and KEGG enrichment analysis were conducted. KEGG pathway enrichment analysis revealed that DEGs were significantly enriched (*p* < 0.05) in multiple metabolic pathways, including plant hormone signal transduction and phenylpropanoid biosynthesis (Figure 4a). Flavonoid biosynthesis is an important branch of phenylpropanoid biosynthesis. The GO enrichment analysis showed that the DEGs were significantly enriched in different GO terms, including 1 term of biological process (BP), 6 terms of cellular components (CC), and 18 terms of molecular function (MF) (for example, transcription factor activity and transcription regulator activity) (Figure 4b).

### 3.4. Analysis of Anthocyanin Biosynthetic Genes

**Figure 5:**
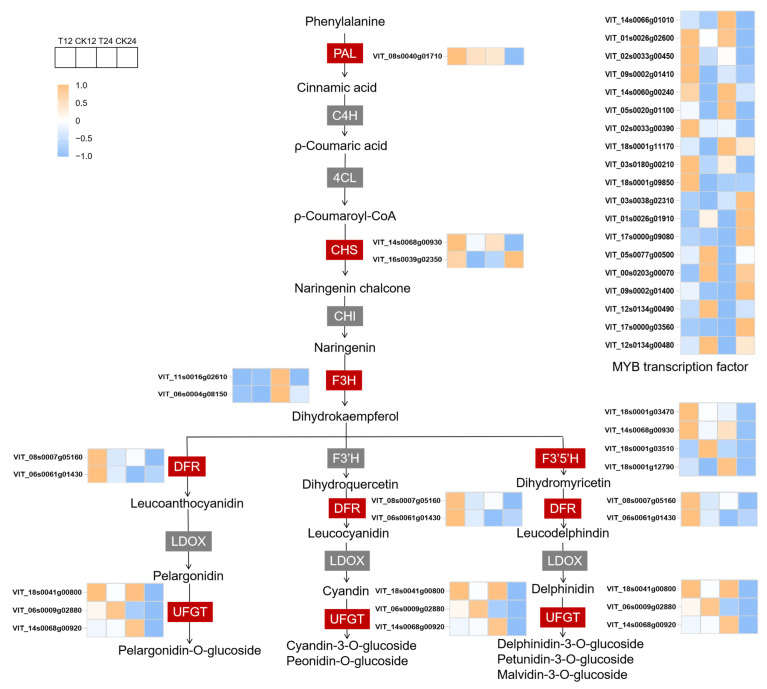
Expression pattern of anthocyanin biosynthetic genes. Gene expression levels are represented by normalized FPKM values. Genes in the red rectangle are expressed differently, and genes in the gray rectangle are not expressed differently.

Based on the transcriptome data, 13 structural genes in the anthocyanin synthesis pathway were screened (Figure 5). *VvPAL* was up-regulated significantly at 24 h after treatment (HAT), and the log_2_FoldChange (log_2_FC) in T24 group was 1.34 (Table S5). ETH treatment induced the most flavonoid biosynthesis genes, including *VvCHS*, *VvF3H*, *VvF3’5*’*H* and *VvDFR*. However, *VvCHI*, *VvF3’H* and *VvLDOX* were not influenced by exogenous ethylene. *VvUFGT* were strongly induced by ETH treatment at 24 HAT (Figure 5, Table S5).

In the EHT-treated berries, 19 MYB genes were expressed differently (*p* < 0.05) (Figure 5, Table S6). *VvMYB98*, *VvMYB105* and *VvMYBA3* were strongly induced by ethylene at 24 HAT (log_2_FC > 1). Meanwhile, the log_2_FC of nine MYB genes were below −1 in the T24 group, indicating that they could play negative regulatory roles in ETH-induced anthocyanin accumulation. VvMYBA1 is an important transcription factor in anthocyanin biosynthesis. In this study, *VvMYBA1* did not express a different response to ethylene, indicating that the anthocyanin accumulation induced by ethylene in the grape berries was independent of VvMYBA1.

**Figure 6:**
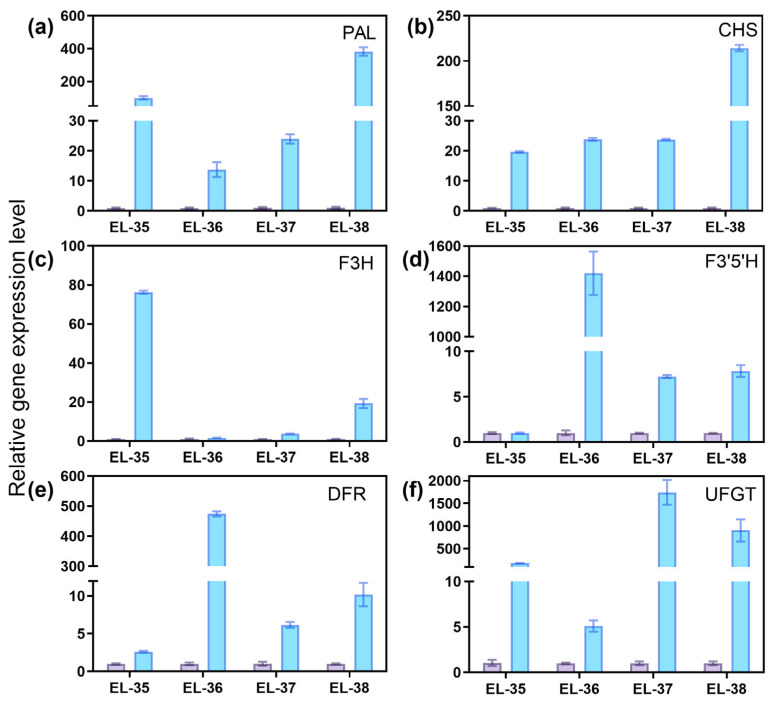
The expression levels of (**a**) PAL, (**b**) CHS, (**c**) F3H, (**d**) F3’5’H, (**e**) DFR and (**f**) UFGT in different phases.

To investigate the long-term effects of exogenous ethylene on the expression of structural genes in anthocyanin biosynthesis, the expression levels of six structural genes were measured at different developmental stages. At EL-38, the expression levels of six genes were all significantly higher in the T group (Figure 6a–f), in which the expression level of *VvUFGT* was the highest, reaching 904 times that of the control (Figure 6f). The expression of *VvUFGT* was even more pronounced at EL-37, reaching 1745 times that of the control (Figure 6f). Additionally, F3’5’H was significantly up-regulated after ETH treatment at EL-36, reaching 1420 times that of the control (Figure 6d). These results suggested that the up-regulation of anthocyanin biosynthetic genes induced by ETH treatment at veraison persisted until berry ripening.

### 3.5. Analysis of Plant Hormone Signal Transduction

**Figure 7:**
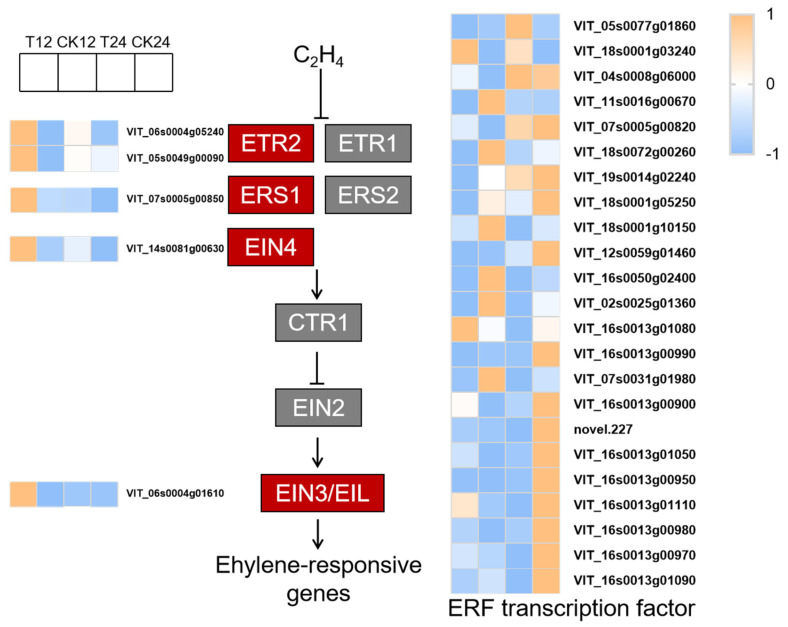
The visualization diagram of the ethylene signaling genes in response to ETH treatment. Gene expression levels are represented by normalized FPKM values.

Ethylene signal transduction genes, *VvETR2*, *VvERS1*, *VvEIN4*, and transcription factor *VvEIL3* showed an increasing trend in response to ETH treatment (Figure 7, Table S7). The ethylene biosynthesis gene, *VvACO*, was up-regulated by ETH treatment (Table S7). In ETH-treated berries, 23 ERF genes were expressed differently compared with the control (*p* < 0.05) (Figure 7, Table S8). *VvERF*12 was induced by exogenous ethylene, but *VvCRF4*, *VvERF61*, *VvABR1* and 5 *VvERF5* genes were strongly suppressed. These results indicate that ERF transcription factors play different regulatory roles in ETH-induced anthocyanin accumulation.

ETH treatment also influenced the biosynthesis and signal transduction of other hormones. The ABA biosynthesis gene, *VvNCED2*, and ABA receptor gene, *VvPYL4*, exhibited an upward trend after ETH treatment (Table S7). However, the zeaxanthin epoxidase gene, *VvZEP*, was repressed at 24 h after treatment. *VvCYP707A* related to ABA catabolism and two ABA transporter genes were up-regulated by ETH treatment (Table S7).

The auxin synthesis gene, *VvTAA3*, and seven genes of the auxin-responsive protein were suppressed by ETH treatment, but the expression level of *VvARF* was increased in ETH-treated berries (Table S7). These results showed that ETH treatment inhibited the biosynthesis and signaling of IAA.

### 3.6. Analysis of Sugar Metabolism

Ethylene treatment promoted the expression of the sugar transporter gene, *VvEDL16*, especially after 24 h of treatment (Table S9). Moreover, two sucrose synthase genes, *VvSUS2* and *VvSUS6*, were induced by ETH treatment (Table S9), accelerating the cleavage of sucrose into hexoses. This was consistent with the increase in Brix in ETH-treated berries.

## 4. Discussion

### 4.1. Effects of Ethylene on Sugar Content and Genes Related to Sugar Metabolism

Anthocyanin biosynthesis is associated with sugar metabolism. In this study, ETH treatment increased Brix in grape berries (Figure 1a), and stimulated the sugar transporter and sucrose synthase genes (Table S9). On one hand, sugars serve as substrates for anthocyanin biosynthesis. It was demonstrated that glucose could be transformed into acyl-glucose, and vacuolar glycosyltransferase (GT) and acyltransferase (AT) could use acyl-glucoses as donor molecules to produce highly modified anthocyanins [19]. On the other hand, sugars serve as signaling molecules for anthocyanin biosynthesis. Sucrose (>2%) induces anthocyanin accumulation in grape berries cultured in vitro, and glucose and fructose exhibit greater efficacy than sucrose [20]. Sugars modulate the expression of both regulatory and structural genes in anthocyanin biosynthesis, particularly in the up-regulation of *VvUFGT* [20].

### 4.2. Effects of Ethylene on Anthocyanin Content, Anthocyanin Biosynthetic Genes and MYB Transcription Factors in Grapes

In this study, the ETH treatment significantly increased the total anthocyanin content and the levels of 21 individual anthocyanins in grape berries (Table S3). Liu et al. (2024) suggested that ETH treatment enhanced anthocyanin and non-anthocyanin biosynthesis in light-exposed grapes, and reduced the ratios of modified to unmodified anthocyanin derivatives [21]. We detected 35 individual anthocyanins in Cabernet Sauvignon, and malvidin 3-O-glucoside was the most abundant. In contrast, nine individual anthocyanins were detected in Muscat Hamburg, and peonidin-3-O-glucoside was the most prevalent [9]. The grape berries were treated with ETH at veraison, and *VvPAl*, *VvCHS*, *VvF3H*, *VvF3’5’H*, *VvDFR* and *VvUFGT* exhibited higher expression in treated berries, not only at 12 or 24 HAT (Figure 5), but also at maturity (EL-38) (Figure 6). Wang et al. (2022) demonstrated that ETH treatment enhanced the expression of anthocyanin structural genes, including *VvPAL*, *VvCHS*, *VvF3H* and *VvGST4*, and the increase was sustained throughout the following 15 days [9]. These results indicate that the influence of ethylene on the expression of anthocyanin biosynthesis genes persisted over an extended period.

MYB transcription factors play important roles in regulating anthocyanin biosynthesis in grape berries. Specifically, VvMYBA1 and VvMYBA2 were identified as vital color regulators in Eurasian grape varieties [22]. There were different results on the effect of ethylene on *VvMYBA1* expression. Some research found that *VvUFGT* induced by ethylene was independent of VvMYBA1 [23]. Other research certified that ethylene treatment strongly induced the expression of *VvMYBA1* [24]. The effects of ethylene on *VvMYBA1* expression remain unclear, and diverse regulatory mechanisms may exist. In this study, *VvUFGT* was up-regulated at 12 h and 24 h after ETH treatment (Table S5), but the transcription level of *VvMYBA1* remained unchanged (Table S5). Zhang et al. (2025) overexpressed *VvMYB24* in grape calli, enhancing anthocyanin accumulation by activating the expression of *VvDFR* and *VvUFGT* [25]. VvMYB24 formed a protein complex with VvMYBA1, promoting the expression of these structural genes. Moreover, VvMYBA1 interacted with VvMYC1 to promote the expression of *VvGT1* and *VvGST4*, which were involved in anthocyanin transport [26].

### 4.3. Effects of Ethylene on Biosynthesis and Signaling of Plant Hormones

The ethylene biosynthesis gene, *VvACO*, was up-regulated by ETH treatment (Figure 7, Table S7). Extensive research suggests that exogenous ethylene treatment increases the content of endogenous ethylene and anthocyanin in non-climacteric fruits, such as grapes [27], strawberries [28] and mulberries [29]. In this study, the genes in ethylene signal transduction, *VvETR2*, *VvERS1*, *VvEIN4* and *VvEIL3*, showed an increasing trend in response to ETH treatment (Figure 7, Table S7). These results indicate that exogenous ethylene promoted endogenous ethylene biosynthesis and signal transduction.

In the process of ethylene signal transduction, EIN3/EIL1 can activate downstream ERF transcription factors, which are essential in anthocyanin synthesis induced by ethylene. In this study, 23 *ERF* genes were expressed differently in ETH-treated berries, in which *VvERF12* was strongly induced (Figure 7, Table S8). On one hand, ERFs may regulate the expression of anthocyanin structural genes. MdERF78 in apple activated the expression of *MdF3H* and *MdANS* [30]; *PsERF3* in plum promoted the expression of *PsANS* [31]. On the other hand, ERFs may synergize with MYB or other transcription factors. CsERF061 in citrus formed a protein complex with CsRuby1 to co-activate the expression of anthocyanin-related genes [32]. MpERF105-MpNAC72 protein complex in Malus promoted the expression of *MpMYB10b* [33]. Additionally, it was shown that VvERF4 in grape physically interacted with histone deacetylase VvHDAC19 to form a complex that bound to the promoter of *VvMYB5a*, thereby synergistically inhibiting the genes in anthocyanin biosynthesis [34]. Currently, reports on ERF-mediated regulation of anthocyanin biosynthesis in grapes remain limited, representing an important direction for future research.

The ABA biosynthesis gene, *VvNCED2*, and ABA receptor gene, *VvPYL4*, were up-regulated in ETH-treated berries (Table S7), indicating that ethylene promoted ABA biosynthesis and signaling. It was demonstrated that ABA promoted anthocyanin accumulation by up-regulating the expression of anthocyanin structural genes [35]. ETH treatment increased ABA content in strawberries by repressing the expression of miR161, which negatively regulated ABA biosynthesis [28]. Exogenous ethylene increased the ABA concentration in blueberries, and induced the expression of the ABA receptor gene, *PYR1-like* [36]. Inversely, blocking ethylene signaling with 1-MCP delayed ABA accumulation [37]. These results suggest that ETH treatment amplifies the ABA signaling, and they exhibit synergistic effects in promoting anthocyanin biosynthesis.

IAA was synthesized through a two-step pathway: tryptophan aminotransferase of arabidopsis1/tryptophan aminotransferase related (TAA1/TAR) converted tryptophan into indole-3-pyruvate, which was then oxidized to IAA by YUCCA monooxygenases [38,39,40]. In this study, auxin synthesis gene *VvTAA3* and seven genes of the auxin-responsive protein were down-regulated in response to ethylene (Table S7). It was demonstrated that IAA treatment decreased the anthocyanin content in red raspberries by inhibiting expression of *MYB10* and *ANS* [41]. These results indicate that ethylene enhanced anthocyanin accumulation by suppressing IAA biosynthesis and signaling.

## 5. Conclusions

ETH treatment increased the content of 21 individual anthocyanins in grape berries, but did not affect the anthocyanin profile. Key structural genes in the anthocyanin synthesis pathway were strongly induced by ETH treatment, including *VvPAL*, *VvCHS*, *VvF3H*, *VvF3′5′H*, *VvDFR* and *VvUFGT*. ETH treatment enhanced sugar transport and biosynthesis, stimulated biosynthesis and signal transduction of ethylene and ABA, but suppressed IAA biosynthesis and signaling. This study provides the theoretical foundation and technical support for the regulation of anthocyanin biosynthesis in non-climacteric fruit.

## Data Availability

The original contributions presented in the study are included in the article/Supplementary Materials, further inquiries can be directed to the corresponding authors.

## Supplementary Materials

The following supporting information can be downloaded at: https://www.mdpi.com/article/10.3390/foods14142551/s1, Table S1: Anthocyanin standard; Table S2: Primer sequences of structural genes in anthocyanin biosynthesis; Table S3: Effects of ETH treatment on the anthocyanin composition in grape berries (μg/g); Table S4: The co-expressed differentially expressed genes (DEGs) in groups T12 and T24; Table S5: Effects of ETH treatment on the structural genes in anthocyanin biosynthesis; Table S6: Effects of ETH treatment on MYB genes; Table S7: Effects of ETH treatment on genes related to plant hormones; Table S8: Effects of ETH treatment on ERF genes; Table S9: Effects of ETH treatment on genes related to sugar metabolism; Figure S1: The chromatograms for different anthocyanins. (a) Total ion chromatogram (TIC). (b) Extracted ion chromatogram (XIC).
