# Supplementary material for: Transcriptome and Anthocyanin Profile Analysis Reveals That Exogenous Ethylene Regulates Anthocyanin Biosynthesis in Grape Berries
Source: Foods. 2025 Jul 21;14(14):2551. doi: 10.3390/foods14142551 (PMC12294778; doi:10.3390/foods14142551)
Supplement: Supplementary file 1 [file foods-14-02551-s001.zip › foods-3726337-supplementary.pdf]

foods

MDPI

Article

Transcriptome and Anthocyanin Profile Analysis Reveals That Exogenous Ethylene Regulates Anthocyanin Biosynthesis in Grape Berries

Min Liu *, Boyuan Fan, Le Li, Jinmei Hao, Ruteng Wei, Hua Luo, Fei Shi, Zhiyuan Ren and Jun Wang

College of Food Science and Engineering, Shanxi Agricultural University, Taigu 030801, China; fby0107@126.com (B.F.); yilelee@126.com (L.L.); hjm1255725667@163.com (J.H.); weiruteng@sxau.edu.cn (R.W.); luohua712@126.com (H.L.); shifei@sxau.edu.cn (F.S.); rzyrzm@gmail.com (Z.R.); wj8154533@126.com (J.W.)

* Correspondence: liumin272@163.com; Tel.: +86-19834544283

Table S1. Anthocyanin standard

Compound	RT	Equation	r
**Cyanidin**			
Cyanidin 3,5-O-diglucoside	4.16	y = 4.89074e4 x + 2908.97570	0.99635
Cyanidin 3-O-(6-O-malonyl-beta-D-glucoside)	8.27	y = 5428.11406 x + 1921.31591	0.99857
Cyanidin 3-O-arabinoside	6.05	y = 1.61840e5 x - 1545.98745	0.99208
Cyanidin 3-O-galactoside	5.06	y = 6.75951e4 x + 12660.47377	0.99811
Cyanidin 3-O-glucoside	5.63	y = 6.61400e4 x + 2668.26662	0.99870
Cyanidin 3-O-rutinoside	6.27	y = 4.89074e4 x + 2908.97570	0.99635
**Delphinidin**			
Delphinidin 3,5-O-diglucoside(Delphin)	3.28	y = 28171.61130 x - 5.57467e4	0.99846
Delphinidin 3-O-(6''-O-malonyl)-beta-D-glucoside	7.2	y = 5428.11406 x + 1921.31591	0.99857
Delphinidin 3-O-arabinoside	5.15	y = 7.48064e4 x - 3.24513e4	0.99653
Delphinidin 3-O-galactoside	4.12	y = 7.48064e4 x - 3.24513e4	0.99653
Delphinidin 3-O-glucoside	4.62	y = 26059.57578 x - 4.83337e4	0.99566
**Malvidin**			
Malvidin 3,5-diglucoside	5.92	y = 3.97020e4 x + 520.19933	0.99671
Malvidin 3-O-(6-O-malonyl-beta-D-glucoside)	10.1	y = 5428.11406 x + 1921.31591	0.99857
Malvidin 3-O-arabinoside	8.32	y = 1.56785e5 x - 65.81059	0.99242
Malvidin 3-O-galactoside	7.39	y = 1.86329e5 x + 13619.80562	0.99153
Malvidin 3-O-glucoside	7.84	y = 1981.76074 x - 426.37155	0.99444
**Pelargonidin**			
Pelargonidin 3-O-glucoside	6.61	y = 1.13319e5 x + 705.72845	0.99453
Pelargonidin 3-O-rutinoside	7.47	y = 4.46537e4 x + 1303.80171	0.99938
**Peonidin**			
Peonidin 3,5-O-diglucoside	5.58	y = 6.35434e4 x + 812.86775	0.99321

 https://doi.org/10.3390/foods14142551

Peonidin 3-O-(6-O-malonyl-beta-D-glucoside)	9.78	y = 5428.11406 x + 1921.31591	0.99857
Peonidin 3-O-arabinoside	7.74	y = 2.98036e5 x - 28.75746	0.99988
Peonidin 3-O-galactoside	6.77	y = 2.79076e5 x + 18486.36364	0.99124
Peonidin 3-O-glucoside	7.32	y = 2.79076e5 x + 18486.36364	0.99124
Peonidin 3-O-rutinoside	7.87	y = 6.35434e4 x + 812.86775	0.99321
**Petunidin**			
Petunidin 3,5-diglucoside	4.72	y = 4.89074e4 x + 2908.97570	0.99635
Petunidin 3-O-(6-O-malonyl-beta-D-glucoside)	8.82	y = 5428.11406 x + 1921.31591	0.99857
Petunidin 3-O-arabinoside	6.87	y = 1.11988e5 x - 11640.39589	0.99906
Petunidin 3-O-galactoside	5.92	y = 1.11988e5 x - 11640.39589	0.99906
Petunidin 3-O-glucoside	6.39	y = 1.11988e5 x - 11640.39589	0.99906
**Others**			
Procyanidin B1	2.07	y = 2352.20689 x + 83.74148	0.99782
Procyanidin B2	3.42	y = 3428.17290 x + 401.41185	0.99979
Procyanidin B3	1.84	y = 3360.17215 x + 59.84607	0.99554
Procyanidin C1	4.58	y = 50.54790 x + 650.48108	0.99772
Quercetin 3-O-glucoside	8.76	y = 3415.41333 x - 581.43504	0.99591
Dihydromyricetin	3.49	y = 855.32775 x - 2577.86189	0.99128

Table S2. Primer sequences of structural genes in anthocyanin biosynthesis.

Gene name	Gene ID	Primer sequence-F	Primer sequence-R
CHS	100263443	GTCTGAAGGAAGA-GAAACTGAGAG	CCAGGATAAACAACAC-GCAT
F3'5'H	100261319	AAACCGCTCAGAC-CAAAACC	ACTAAGCCACAG-GAAACTAA
F3H	100233079	CTGTGGTGAACTCCGACTGC	CAAATGTTATGGGCTCCTC C
DFR	100233141	GGCCAAATCAAACTAC-CAGA	GAAACCTGTAGATGG-CAGGA
PAL	100245997	CAACCAA-GATGTGAACTCCTT	TTCTCCTCCAAATGCCTC
UFGT	100233099	GGGATGGTAATGGCTGTGG	ACATGGGTGGAGAG-TGAGTT
Actin	100246726	CTTGCATCCCTCAGCACCTT	TCCTGTG-GACAATGGATGGA

Table S3. Effects of ETH treatment on the anthocyanin composition in grape berries (μg/g).

Compound	CK	Treatment
**Cyanidin**		
Cyanidin 3,5-O-diglucoside	0.11±0.01**	0.15±0.03**
Cyanidin 3-O-(6-O-malonyl-beta-D-glucoside)	0.03±0.001**	0.02±0.001**
Cyanidin 3-O-arabinoside	0.002±0.0002**	0.003±0.0004**

Cyanidin 3-O-galactoside	0.17±0.01	0.16±0.02
Cyanidin 3-O-glucoside	38.27±2.17**	42.6±7.19**
Cyanidin 3-O-rutinoside	0.01±0.002*	0.015±0.001*
**Delphinidin**		
Delphinidin 3,5-O-diglucoside(Delphin)	0.21±0.01*	0.18±0.004*
Delphinidin 3-O-(6''-O-malonyl)-beta-D-glucoside	0.06±0.01	0.05±0.01
Delphinidin 3-O-arabinoside	0.02±0.0003**	0.02±0.001**
Delphinidin 3-O-galactoside	0.80±0.01**	0.68±0.02**
Delphinidin 3-O-glucoside	290.67±5.13**	305.67±17.90**
**Malvidin**		
Malvidin 3,5-diglucoside	0.72±0.04	0.74±0.05
Malvidin 3-O-(6-O-malonyl-beta-D-glucoside)	3.89±0.06	3.84±0.35
Malvidin 3-O-arabinoside	0.04±0.003	0.04±0.004
Malvidin 3-O-galactoside	6.71±0.19	7.09±0.20
Malvidin 3-O-glucoside	375.33±20.55**	446±33**
**Pelargonidin**		
Pelargonidin 3-O-glucoside	0.71±0.06**	0.88±0.17**
Pelargonidin 3-O-rutinoside	0.09±0.01	0.09±0.01
**Peonidin**		
Peonidin 3,5-O-diglucoside	0.26±0.01**	0.34±0.05**
Peonidin 3-O-(6-O-malonyl-beta-D-glucoside)	0.14±0.01**	0.16±0.03**
Peonidin 3-O-arabinoside	0.003±0.00	0.003±0.0001
Peonidin 3-O-galactoside	1.14±0.04**	1.33±0.08**
Peonidin 3-O-glucoside	29.27±0.06*	32.4±2.71*
Peonidin 3-O-rutinoside	0.06±0.002	0.07±0.01
**Petunidin**		
Petunidin 3,5-diglucoside	0.03±0.0002**	0.04±0.001**
Petunidin 3-O-(6-O-malonyl-beta-D-glucoside)	0.09±0.01	0.08±0.01
Petunidin 3-O-arabinoside	0.02±0.001	0.02±0.002
Petunidin 3-O-galactoside	0.44±0.01	0.46±0.03
Petunidin 3-O-glucoside	67.23±5.59*	72.63±4.05*
**Others**		
Procyanidin B1	195.33±15.37**	202.33±2.52**
Procyanidin B2	127.67±13.87**	135.3333±7.02**
Procyanidin B3	99.6±7.28**	101.6±2.62**
Procyanidin C1	50.13±5.75**	54±3.92**
Quercetin 3-O-glucoside	64.77±11.28	46.03±13.15
Dihydromyricetin	0.56±0.04	0.58±0.06
**Total**	1354.60±3.48**	1455.63±65.75 **

Table S4. The co-expressed differentially expressed genes (DEGs) in groups of T12 and T24.

Gene ID	T24 vs CK24		T12 vs CK12		Gene description
	log_2FC	pvalue	log_2FC	pvalue	
VIT_09s0002g05160	-0.95	0.0010	-0.65	0.0039	Auxin-responsive protein IAA17
VIT_16s0050g01900	-2.49	0.0125	-2.26	0.0470	Receptor-like protein EIX1
VIT_16s0100g00800	-2.99	0.0157	1.71	0.0233	Stilbene synthase 4
VIT_05s0020g02810	0.57	0.0459	0.76	0.0040	Protein trichome birefringence-like 10
VIT_00s0370g00020	0.41	0.0078	0.52	0.0092	Clathrin light chain 1

VIT_03s0038g03570	0.85	0.0001	0.59	0.0045	L-ascorbate oxidase homolog
VIT_10s0003g01170	-3.00	0.0000	1.20	0.0387	Transcription factor bHLH30
VIT_11s0052g00100	-0.93	0.0124	-0.94	0.0301	Transcription factor bHLH35
VIT_14s0068g00010	-0.97	0.0118	-1.27	0.0077	Receptor-like protein kinase FERONIA
VIT_14s0068g02150	0.60	0.0291	1.01	0.0006	F-box/kelch-repeat protein
VIT_16s0039g01330	-1.61	0.0136	-1.53	0.0256	Rust resistance kinase Lr10
VIT_16s0050g00570	0.66	0.0238	0.97	0.0038	Pectin acetylesterase 9
VIT_18s0001g13790	0.65	0.0122	0.93	0.0100	Cytochrome P450 83B1
VIT_00s0480g00070	-2.00	0.0000	-1.31	0.0045	Polyphenol oxidase, chloroplastic
VIT_10s0116g00560	-1.99	0.0000	-1.31	0.0001	Polyphenol oxidase, chloroplastic
VIT_11s0016g01220	-1.53	0.0001	-0.84	0.0341	Auxin-binding protein ABP19a
VIT_17s0000g09760	1.10	0.0297	1.50	0.0021	Putative multidrug resistance protein
VIT_18s0001g04150	0.77	0.0027	0.56	0.0494	Beta-1,3-galactosyltransferase 7
VIT_00s0309g00050	-4.90	0.0101	3.70	0.0027	Expansin-like B1
VIT_01s0011g04770	0.51	0.0236	0.57	0.0140	U-box domain-containing protein 11
VIT_14s0083g00370	0.95	0.0280	2.46	0.0000	Protein trichome birefringence-like 19
VIT_04s0023g01080	0.64	0.0406	0.96	0.0441	LysM domain receptor-like kinase 4
VIT_13s0019g04030	0.57	0.0045	0.48	0.0300	Serine carboxypeptidase-like 27
VIT_05s0094g01080	-1.94	0.0008	-0.88	0.0361	Integrin-linked protein kinase 1
VIT_06s0004g06850	0.90	0.0021	1.24	0.0000	Mitogen-activated protein kinase kinase kinase 17
VIT_14s0036g00420	-0.61	0.0480	0.78	0.0073	Phosphoenolpyruvate carboxylase kinase 1
VIT_00s0203g00210	0.55	0.0168	-0.63	0.0122	-
VIT_10s0003g05420	1.33	0.0087	1.20	0.0081	Berberine bridge enzyme-like 15
VIT_17s0000g06300	0.69	0.0086	0.91	0.0074	-
VIT_17s0000g07400	-2.45	0.0088	-2.90	0.0044	Protein EDS1L
VIT_09s0002g00570	-1.06	0.0040	-0.91	0.0172	GDSL esterase/lipase 1
VIT_11s0016g03350	-2.26	0.0000	-1.57	0.0347	Dehydration-responsive element-binding protein 3
VIT_11s0052g01200	2.07	0.0047	2.39	0.0017	Probable xyloglucan endotransglucosylase/hydrolase protein 23
VIT_11s0052g01270	1.77	0.0005	1.33	0.0353	Probable xyloglucan endotransglucosylase/hydrolase protein 23
VIT_16s0013g00900	-2.09	0.0157	2.70	0.0087	Ethylene-responsive transcription factor 5

VIT_16s0013g01080	-1.58	0.0012	0.77	0.0382	Ethylene-responsive transcription factor 5
VIT_18s0001g03240	0.88	0.0264	1.10	0.0002	Ethylene-responsive transcription factor ERF12
VIT_18s0001g10150	-0.52	0.0452	-0.79	0.0106	Ethylene-responsive transcription factor ERF10
VIT_05s0020g04420	-1.72	0.0262	-1.96	0.0138	Calmodulin-3
VIT_03s0063g01690	-1.50	0.0004	-1.98	0.0000	Xanthotoxin 5-hydroxylase CYP82C4
VIT_06s0004g00410	-0.49	0.0096	-0.43	0.0140	Neprosin|PF14365:Neprosin activation peptide
VIT_13s0019g03610	1.12	0.0037	2.44	0.0003	Probable RNA-binding protein ARP1
VIT_16s0039g01900	0.53	0.0394	0.87	0.0400	Probable transcription factor KAN2
VIT_19s0014g04790	0.39	0.0251	0.92	0.0001	Organic cation/carnitine transporter 4
VIT_08s0007g08330	0.71	0.0155	0.96	0.0000	Polygalacturonase
VIT_13s0019g02300	-0.76	0.0433	-0.73	0.0053	Wall-associated receptor kinase-like 20
VIT_18s0001g06090	-3.87	0.0000	-5.14	0.0040	7-deoxyloganetin glucosyltransferase
VIT_18s0001g09360	-1.01	0.0007	-1.09	0.0041	Subtilisin-like protease SBT1.7
VIT_03s0088g00620	0.42	0.0112	0.53	0.0167	PF06219:Protein of unknown function (DUF1005)
VIT_11s0016g02920	0.83	0.0069	0.86	0.0083	PF06219:Protein of unknown function (DUF1005)
VIT_13s0156g00110	0.73	0.0005	-1.29	0.0276	ATPase
VIT_19s0014g02190	0.55	0.0212	1.03	0.0407	Probable aminotransferase TAT2
VIT_00s0203g00070	-3.56	0.0002	-2.96	0.0250	Transcription factor MYB102
VIT_02s0033g00450	1.04	0.0221	0.99	0.0003	Transcription factor MYB1
VIT_07s0005g02450	-0.82	0.0081	-0.53	0.0160	-
VIT_12s0134g00490	-4.99	0.0055	-2.19	0.0476	Transcription factor MYB4
VIT_17s0000g01970	-1.20	0.0000	0.68	0.0261	Mitogen-activated protein kinase kinase 9
VIT_18s0001g06150	1.43	0.0002	1.12	0.0130	Protein PHOSPHATE-INDUCED 1
VIT_18s0001g11930	0.47	0.0296	0.33	0.0282	Pathogenesis-related thaumatin-like protein 3.5
VIT_19s0015g01300	0.51	0.0376	0.54	0.0008	Probable amino acid permease 7
VIT_04s0008g01500	2.17	0.0363	1.79	0.0331	17.3 kDa class II heat shock protein
VIT_04s0008g01530	1.17	0.0119	0.82	0.0366	17.3 kDa class II heat shock protein
VIT_04s0023g02290	-0.85	0.0042	-0.87	0.0013	S-adenosyl-L-methionine:benzoic acid/salicylic acid carboxyl methyltransferase 3

VIT_04s0044g01560	-0.57	0.0275	-0.87	0.0001	Proline-rich receptor-like protein kinase PERK9
VIT_05s0077g01760	0.56	0.0097	0.84	0.0060	Probable polygalacturonase
VIT_06s0061g01470	1.23	0.0013	1.72	0.0000	Pleiotropic drug resistance protein 2
VIT_11s0052g01260	1.67	0.0003	1.48	0.0114	Probable xyloglucan endo-transglucosylase/hydrolase protein 23
VIT_13s0158g00360	1.39	0.0000	1.15	0.0224	Putative calcium-transporting ATPase 11, plasma membrane-type
VIT_14s0108g01020	-1.49	0.0000	-2.27	0.0000	Expansin-A10
VIT_18s0001g08580	0.79	0.0012	0.74	0.0115	Probable xyloglucan glycosyltransferase 12
VIT_00s0153g00050	0.96	0.0000	1.17	0.0005	Probable glutathione S-transferase
VIT_03s0038g03410	-2.96	0.0006	-1.64	0.0114	NAC domain-containing protein 6
VIT_04s0044g01020	-0.70	0.0486	-0.96	0.0413	Probable pectinesterase/pectinesterase inhibitor 51
VIT_06s0004g00990	-0.90	0.0426	-0.51	0.0260	Dirigent protein 19
VIT_08s0007g08120	0.79	0.0026	0.91	0.0001	Probable glucuronoxylan glucuronosyltransferase F8H
VIT_10s0003g01420	0.49	0.0090	0.51	0.0085	CBL-interacting serine/threonine-protein kinase 12
VIT_11s0052g01190	1.73	0.0010	1.50	0.0142	Probable xyloglucan endo-transglucosylase/hydrolase protein 23
VIT_12s0035g00880	-1.23	0.0000	-0.58	0.0143	PF00903:Glyoxalase/Bleomycin resistance protein/Dioxygenase superfamily
VIT_13s0158g00080	-1.40	0.0228	-1.41	0.0024	Serine carboxypeptidase-like 40
VIT_14s0108g00840	-1.06	0.0254	-1.21	0.0363	PF07891:Protein of unknown function (DUF1666)
VIT_11s0016g01530	-4.02	0.0000	2.18	0.0373	Ankyrin repeats
VIT_04s0008g01580	0.80	0.0218	1.37	0.0006	17.3 kDa class II heat shock protein
VIT_16s0100g00570	0.47	0.0447	0.59	0.0056	Probable methyltransferase PMT15
VIT_12s0055g00810	0.70	0.0054	-0.68	0.0233	Peroxidase 43
VIT_03s0167g00050	1.97	0.0023	5.19	0.0059	L-type lectin-domain containing receptor kinase IX.1
VIT_04s0008g00310	-5.45	0.0001	-2.23	0.0252	Leucine-rich repeat receptor-like kinase protein CLV1a
VIT_16s0050g01190	-4.18	0.0004	-1.37	0.0294	Heavy metal-associated isoprenylated plant protein 39
VIT_02s0025g02970	-1.10	0.0059	-1.09	0.0102	Protein DMR6-LIKE OXYGENASE 2
VIT_03s0063g01210	2.70	0.0001	3.03	0.0000	Jasmonate-induced oxygenase 2

VIT_05s0020g01310	-1.36	0.0269	1.59	0.0134	Protein SRG1
VIT_07s0005g02710	1.02	0.0106	0.59	0.0076	-
VIT_11s0016g00590	-0.44	0.0275	-0.72	0.0038	Pectinesterase inhibitor 3
VIT_13s0019g01240	0.39	0.0205	0.64	0.0058	Actin-1
VIT_04s0044g01300	1.34	0.0004	1.20	0.0004	5' exonuclease Apollo
VIT_15s0021g00890	-0.67	0.0202	-0.55	0.0099	E3 ubiquitin-protein ligase ATL4
VIT_02s0025g02960	-0.99	0.0000	-1.55	0.0024	Protein DMR6-LIKE OXYGENASE 2
VIT_04s0069g00920	-1.32	0.0000	-1.12	0.0368	Probable WRKY transcription factor 11
VIT_07s0031g01710	-1.96	0.0231	-1.69	0.0160	Probable WRKY transcription factor 51
VIT_08s0040g02730	1.01	0.0028	1.20	0.0001	Exocyst complex component EXO70H1
VIT_10s0003g02810	-2.43	0.0030	-2.75	0.0238	WRKY transcription factor 28
VIT_06s0004g01140	0.71	0.0104	0.74	0.0115	Serine/threonine-protein kinase GRIK1
VIT_13s0106g00230	-3.59	0.0000	-1.10	0.0242	PF12796:Ankyrin repeats
VIT_00s0187g00090	-0.93	0.0465	-1.17	0.0300	PF07430:Phloem filament protein PP1 cystatin-like domain
VIT_00s0587g00030	0.61	0.0018	0.88	0.0005	CBS domain-containing protein CBSX5
VIT_01s0010g00760	-3.82	0.0097	-2.54	0.0159	-
VIT_01s0011g04370	0.74	0.0078	0.61	0.0388	CDP-diacylglycerol-serine O-phosphatidyltransferase 1
VIT_01s0011g05190	-0.87	0.0046	-0.88	0.0452	MLP-like protein 28
VIT_01s0011g05950	1.05	0.0005	1.32	0.0002	MACPF domain-containing protein At1g14780
VIT_01s0146g00480	-1.99	0.0031	-1.44	0.0299	Protein TIFY 9
VIT_02s0025g04870	1.11	0.0016	1.69	0.0000	-
VIT_02s0033g01400	0.81	0.0212	1.36	0.0000	Ubiquinol oxidase 1a, mitochondrial
VIT_03s0038g01510	-1.92	0.0000	-0.71	0.0170	-
VIT_03s0091g00410	0.46	0.0189	-0.70	0.0205	PF04788:Protein of unknown function (DUF620)
VIT_04s0008g00860	0.50	0.0394	1.25	0.0005	Protein NDL2
VIT_04s0008g01700	1.19	0.0410	1.97	0.0038	Protein BIG GRAIN 1-like B
VIT_04s0008g04150	-1.11	0.0442	-1.48	0.0108	BURP domain protein RD22
VIT_04s0008g05470	-0.65	0.0015	-0.84	0.0001	EIN3-binding F-box protein 2
VIT_04s0023g02150	0.59	0.0009	0.99	0.0000	Lipid phosphate phosphatase epsilon 2, chloroplastic
VIT_04s0023g03170	0.54	0.0098	0.47	0.0034	GDP-mannose 4,6 dehydratase 1
VIT_04s0044g00220	0.61	0.0100	0.51	0.0340	Monooxygenase 2
VIT_05s0020g00330	-2.94	0.0084	-2.69	0.0135	Galactinol synthase 2
VIT_05s0049g01100	-1.22	0.0353	-1.64	0.0493	Probable glutathione S-transferase
VIT_05s0077g00540	0.80	0.0357	3.14	0.0000	-
VIT_05s0102g00190	-4.45	0.0001	4.01	0.0000	-

VIT_06s0004g04640	-1.12	0.0116	-1.33	0.0046	PF01439:Metallothionein
VIT_06s0004g05240	1.21	0.0298	2.02	0.0038	Ethylene receptor 2
VIT_06s0004g05680	-0.72	0.0085	-0.78	0.0023	Glutathione S-transferase U7
VIT_08s0007g05580	0.95	0.0025	0.56	0.0392	Putative methyltransferase DDB_G0268948
VIT_08s0007g08010	0.71	0.0015	0.89	0.0000	-
VIT_08s0007g08020	1.31	0.0002	1.41	0.0013	-
VIT_08s0032g00930	0.46	0.0073	0.47	0.0132	Fasciclin-like arabinogalactan protein 17
VIT_08s0058g00860	-0.90	0.0279	-1.51	0.0106	Protein LURP-one-related 15
VIT_10s0003g01020	1.95	0.0001	0.88	0.0473	Adenylate-forming reductase
VIT_10s0003g02680	-0.85	0.0123	-0.68	0.0186	Phytochrome E
VIT_10s0116g00370	0.51	0.0083	0.53	0.0214	-
VIT_11s0016g03900	0.79	0.0001	0.58	0.0164	Ribulose bisphosphate carboxylase/oxygenase activase, chloroplastic (strain 10D)
VIT_11s0016g05530	0.58	0.0082	0.78	0.0036	Plastocyanin-like domain
VIT_11s0103g00520	-3.61	0.0000	-1.95	0.0244	Domain of unknown function (DUF4228)
VIT_12s0034g01970	0.63	0.0347	0.67	0.0402	Cupin
VIT_12s0035g02080	-1.10	0.0092	-0.77	0.0050	START domain-containing protein 10
VIT_12s0057g00950	-2.75	0.0008	-2.52	0.0020	-
VIT_13s0067g00990	-0.46	0.0391	-1.03	0.0086	Protein SMAX1-LIKE 7
VIT_13s0074g00110	1.14	0.0013	0.98	0.0035	Bark storage protein A
VIT_14s0006g01600	-1.67	0.0001	-1.92	0.0069	-
VIT_14s0060g01730	-0.80	0.0021	-0.65	0.0438	-
VIT_14s0083g00520	1.28	0.0138	1.55	0.0000	Proline dehydrogenase 1, mitochondrial
VIT_15s0024g00410	-2.39	0.0303	-2.17	0.0418	Uncharacterized protein PHLOEM PROTEIN 2-LIKE A4
VIT_16s0050g00410	-4.61	0.0037	-4.73	0.0077	PF06521:PAR1 protein
VIT_18s0001g06560	0.63	0.0052	0.63	0.0092	-
VIT_18s0001g09140	-0.54	0.0272	-0.51	0.0077	Probable strigolactone esterase DAD2
VIT_18s0001g13610	0.33	0.0472	0.37	0.0471	1-acylglycerol-3-phosphate O-acyltransferase
VIT_19s0090g00120	1.89	0.0005	1.26	0.0002	Fatty acid desaturase 4, chloroplastic
VIT_19s0090g00790	-4.46	0.0262	-3.63	0.0489	Probable F-box protein
novel.222	-0.57	0.0498	-1.29	0.0008	-
novel.290	-1.04	0.0045	-0.87	0.0075	-
novel.58	0.89	0.0076	0.36	0.4448	-

Table S5. Effects of ETH treatment on the structural genes in anthocyanin biosynthesis.

Gene ID	T24 vs CK24		T12 vs CK12		Gene description
	log_2FC	pvalue	log_2FC	pvalue	

VIT_08s0040g01710	1.34	0.0033	0.29	0.4071	phenylalanine ammonial-yase PAL
VIT_14s0068g00930	1.15	0.0034	0.61	0.1320	chalcone synthase CHS
VIT_16s0039g02350	-0.62	0.4153	0.72	0.4100	chalcone synthase CHS
VIT_11s0016g02610	6.47	0.0980	-1.81	0.6537	flavanone 3-hydroxylase F3H
VIT_06s0004g08150	0.50	0.1610	0.05	0.7900	flavanone 3-hydroxylase F3H
VIT_18s0001g03470	1.35	0.0552	0.76	0.2376	Flavonoid 3′,5′-hydroxylase 1 F3'5'H1
VIT_18s0001g03510	0.58	0.5590	-1.61	0.0600	Flavonoid 3′,5′-hydroxylase 1 F3'5'H1
VIT_18s0001g12790	2.86	0.2432	1.42	0.6668	Flavonoid 3′,5′-hydroxylase 2 F3'5'H2
VIT_08s0007g05160	1.68	0.1107	1.12	0.2104	dihydroflavonol reductase DFR
VIT_06s0061g01430	-0.25	0.4640	0.61	0.0800	dihydroflavonol reductase DFR
VIT_18s0041g00800	1.97	0.6253	0.97	0.8107	UDPGlucose: flavonoid 3-O-glucosyltransferase UFGT
VIT_06s0009g02880	0.83	0.8323	-0.75	0.7100	UDPGlucose: flavonoid 3-O-glucosyltransferase UFGT
VIT_14s0068g00920	0.78	0.2484	-0.03	0.9000	UDPGlucose: flavonoid 3-O-glucosyltransferase UFGT

Table S6. Effects of ETH treatment on *MYB* genes.

**Gene ID**	**T24 vs CK24**		**T12 vs CK12**		**Gene description**
	log_2FC	pvalue	log_2FC	pvalue	
**VIT_14s0066g01010**	5.13	0.0209	1.44	0.6554	Transcription factor MYB98
VIT_01s0026g02600	2.22	0.0352	0.72	0.5213	Transcription factor MYB105
VIT_02s0033g00450	1.04	0.0221	0.99	0.0003	Transcription factor MYBA3
VIT_09s0002g01410	0.99	0.3286	2.84	0.0134	Transcription factor MYB1
VIT_14s0060g00240	0.92	0.0019	1.48	0.1746	Transcription factor MYB61
VIT_05s0020g01100	0.83	0.0219	0.41	0.3152	Transcription factor MYB61
VIT_02s0033g00390	0.66	0.1440	0.62	0.0293	Transcription factor MYBA2
VIT_18s0001g11170	0.50	0.2099	1.84	0.0419	Transcription factor MYB73
VIT_03s0180g00210	0.44	0.1181	0.49	0.0299	Transcription factor MYB73
VIT_18s0001g09850	0.00	0.9822	0.69	0.0027	Transcription factor MYB73
VIT_03s0038g02310	-0.57	0.0190	0.13	0.5381	Transcription factor MYB308
VIT_01s0026g01910	-1.01	0.0122	-0.60	0.1914	Transcription factor MYB88
VIT_17s0000g09080	-1.77	0.0424	0.21	0.8481	Transcription factor MYB86

VIT_05s0077g00500	-2.01	0.0117	-1.14	0.1828	Transcription factor MYB108
VIT_00s0203g00070	-3.56	0.0002	-2.96	0.0250	Transcription factor MYB102
VIT_09s0002g01400	-4.08	0.0243	1.97	0.4319	Transcription factor MYB123
VIT_12s0134g00490	-4.99	0.0055	-2.19	0.0476	Transcription factor MYB4
VIT_17s0000g03560	-5.33	0.0049	0.89	0.8066	Transcription factor MYB62
VIT_12s0134g00480	-6.09	0.0226	-1.61	0.2164	Transcription factor MYB4

Table S7. Effects of ETH treatment on genes related to plant hormone.

**Gene ID**	**T24 vs CK24**		**T12 vs CK12**		**Gene description**
	log_2FC	pvalue	log_2FC	pvalue	
**VIT_00s2086g00010**	0.89	0.0642	-0.26	0.6209	1-aminocyclopropane-1-carboxylate oxidase ACO
VIT_14s0081g00630	0.27	0.2458	0.52	0.0204	Ethylene-insensitive protein 4 EIN4
VIT_06s0004g01610	0.04	0.8546	1.17	0.0001	Ethylene insensitive like protein 3 EIL3
VIT_06s0004g05240	1.21	0.0298	2.02	0.0038	Ethylene receptor 2 ETR2
VIT_05s0049g00090	0.16	0.3875	1.72	0.0001	Ethylene receptor 2 ETR2
VIT_07s0005g00850	0.32	0.2739	0.83	0.0006	Ethylene response sensor 1 ERS1
VIT_08s0058g00470	1.77	0.0001	0.76	0.6159	Abscisic acid receptor PYL4
VIT_10s0003g03750	-0.47	0.0617	0.77	0.0332	9-cis-epoxycarotenoid dioxygenase NCED2
VIT_07s0031g00620	-0.81	0.0111	-0.24	0.5152	Zeaxanthin epoxidase ZEP
VIT_02s0087g00710	0.63	0.3419	1.36	0.0007	Abscisic acid 8'-hydroxylase CYP707A2
VIT_03s0017g01280	0.01	0.9779	1.03	0.0018	ABC transporter G family member ABCG
VIT_06s0061g01470	1.23	0.0013	1.72	0.0000	ABC transporter G family member ABCG
VIT_18s0157g00090	-1.49	0.0027	-0.71	0.2207	Tryptophan aminotransferase-related protein 3 TAA3
VIT_15s0048g02860	-0.80	0.0067	-0.09	0.7879	Auxin-responsive protein SAUR32
VIT_02s0154g00010	-2.69	0.0076	-1.69	0.1570	Auxin-responsive protein SAUR36
VIT_09s0002g05160	-0.95	0.0010	-0.65	0.0039	Auxin-responsive protein IAA17
VIT_18s0001g08090	-0.28	0.2172	-0.44	0.0200	Auxin-responsive protein IAA9
VIT_09s0002g04080	-2.83	0.0002	-0.48	0.5311	Auxin-responsive protein IAA27
VIT_11s0016g03540	-2.56	0.0001	-0.92	0.0881	Auxin-responsive protein IAA27
VIT_01s0146g00180	-5.91	0.0001	-0.59	0.7078	Auxin responsive protein
VIT_10s0003g04100	-0.12	0.6854	0.62	0.0400	Auxin response factor ARF

Table S8. Effects of ETH treatment on *ERF* genes.

Gene ID	T24 vs CK24		T12 vs CK12		Gene description
	log_2FC	pvalue	log_2FC	pvalue	
**VIT_05s0077g01860**	0.96	0.0483	-0.14	0.8931	Ethylene-responsive transcription factor RAP-3
VIT_18s0001g03240	0.88	0.0264	1.10	0.0002	Ethylene-responsive transcription factor ERF12
VIT_04s0008g06000	0.10	0.6176	0.79	0.0072	Ethylene-responsive transcription factor ERF3
VIT_11s0016g00670	0.08	0.9245	-2.23	0.0052	Ethylene-responsive transcription factor ERF16
VIT_07s0005g00820	-0.18	0.3305	0.83	0.0067	Ethylene-responsive transcription factor ERF71
VIT_18s0072g00260	-0.23	0.5346	-0.82	0.0292	Ethylene-responsive transcription factor ERF110
VIT_19s0014g02240	-0.24	0.5778	-1.16	0.0067	Ethylene-responsive transcription factor ERF78
VIT_18s0001g05250	-0.49	0.0058	-0.60	0.1006	Ethylene-responsive transcription factor RAP24
VIT_18s0001g10150	-0.52	0.0452	-0.79	0.0106	Ethylene-responsive transcription factor ERF10
VIT_12s0059g01460	-0.65	0.0193	-0.06	0.7772	Ethylene-responsive transcription factor ERF3
VIT_16s0050g02400	-0.71	0.2174	-2.05	0.0165	CRF4 Ethylene-responsive transcription factor CRF4
VIT_02s0025g01360	-0.79	0.0541	-1.46	0.0012	Ethylene-responsive transcription factor ERF61
VIT_16s0013g01080	-1.58	0.0012	0.77	0.0382	Ethylene-responsive transcription factor ERF5
VIT_16s0013g00990	-1.69	0.0349	-0.22	0.8196	Ethylene-responsive transcription factor ERF5
VIT_07s0031g01980	-2.09	0.1211	-3.13	0.0115	Ethylene-responsive transcription factor ABR1
VIT_16s0013g00900	-2.09	0.0157	2.70	0.0087	Ethylene-responsive transcription factor ERF5
novel.227	-2.23	0.0298	0.19	0.8801	Ethylene-responsive transcription factor ERF5
VIT_16s0013g01050	-2.29	0.0091	1.40	0.1097	Ethylene-responsive transcription factor ERF5
VIT_16s0013g00950	-2.36	0.0086	0.12	0.9157	Ethylene-responsive transcription factor ERF5
VIT_16s0013g01110	-2.49	0.0042	1.63	0.0705	Ethylene-responsive transcription factor ERF5

VIT_16s0013g00980	-2.82	0.0152	2.27	0.1388	Ethylene-responsive transcription factor ERF5
VIT_16s0013g00970	-3.97	0.0062	0.54	0.6577	Ethylene-responsive transcription factor ERF5
VIT_16s0013g01090	-4.55	0.0283	-0.96	0.6976	Ethylene-responsive transcription factor ERF5

Table S9. Effects of ETH treatment on genes related to sugar metabolism.

**Gene ID**	**T24 vs CK24**		**T12 vs CK12**		**Gene description**
	log_2FC	pvalue	log_2FC	pvalue	
**VIT_05s0020g02170**	1.92	0.0326	0.65	0.1307	Sugar transporter EDL16
VIT_05s0077g01930	9.20	0.0010	-0.80	0.5765	Sucrose synthase SUS2
VIT_04s0079g00230	2.98	0.0065	-0.97	0.3862	Sucrose synthase SUS6

(a)

(b)

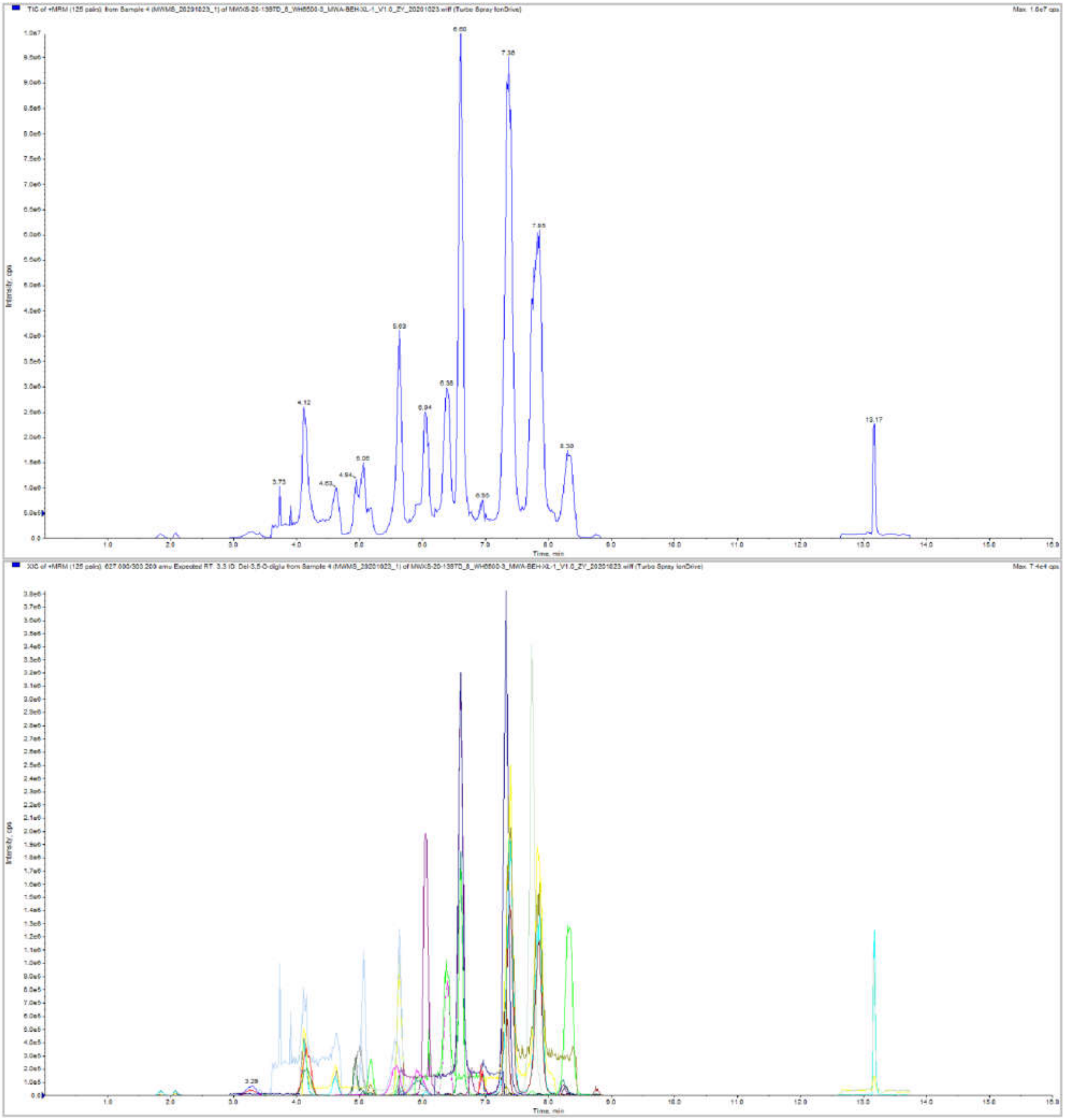

Figure S1. The chromatograms for different anthocyanins. (a) total ion chromatogram (TIC). (b) extracted ion chromatogram (XIC).
